# Associations between the dietary inflammatory index and urinary incontinence among women younger than 65 years

**DOI:** 10.1038/s41598-021-88833-0

**Published:** 2021-04-29

**Authors:** Shiyu Zhang, Haiyang Bian, Shi Qiu, Boyu Cai, Kun Jin, Xiaonan Zheng, Jiakun Li, Xiang Tu, Jianzhong Ai, Lu Yang, Qiang Wei

**Affiliations:** 1 Department of Urology, Institute of Urology and National Clinical Research Center for Geriatrics, West China Hospital of Sichuan University, No. 37, Guoxue Alley, Chengdu, Sichuan 610041 People’s Republic of China; 2 Institute of Reproductive and Child Health and Department of Epidemiology and Biostatistics, Peking University School of Public Health, Beijing, China; 3 Center of Biomedical Big Data, West China Hospital, Sichuan University, Chengdu, Sichuan China

**Keywords:** Diseases, Urology

## Abstract

The purpose of this study was to evaluate the association between dietary inflammatory index (DII) and urinary incontinence (UI) among a representative sample of the US women. We performed a cross-sectional analysis of women younger than 65 years using the 1999 to 2016 NHANES (National Health and Nutrition Examination Survey) populations. DII were calculated based on baseline dietary intake using 24-h dietary recalls. UI was determined and categorized by self-reported questions. Multivariable logistic regression models were used to assess the association between DII and UI. Stratified linear regression models were applied to test for interaction in prespecified subgroup of interest. A total of 13,441 women age between 20 and 65 years were included in the final analysis. Of these participants 3230 (24.03%) complained of urgency UI, 5276 (39.25%) complained of stress UI and 2028 (15.09%) complained of mixed UI. On multivariate analysis, analysis with DII categorized as quartiles revealed significantly increase odds of urgency UI in the most pro-inflammatory quartile compared to the most anti-inflammatory quartile (OR 1.24, 95% CI 1.07–1.44, P = 0.004 for trend) in full adjustment model. Similar results were observed in SUI (OR 1.14, 95% CI 1.00–1.30, P = 0.021 for trend) and MUI (OR 1.20, 95% CI 1.02−1.43, P = 0.022 for trend). More pro-inflammatory diets, as presented by higher DII scores are associated with an increased likelihood of UI in American women younger than 65 years. Further studies are needed to explore the possible physiological mechanism and evaluate the potential therapeutic implications.

## Introduction

Urinary incontinence (UI) is a common complaint that afflicts 17.1% of women aged 20 years or older according to a cross-sectional evidence^1^ and the prevalence increases with age^2^. It decreases quality of life, as well as increases significant costs, to both individuals and societies. UI commonly presents as either stress UI, urgency UI, or mixed UI.

Diet, as mixture of pro-inflammatory or anti-inflammatory compounds, is an important potential source and modulator of inflammation^3^. The dietary inflammatory index (DII) was developed to estimate an individual’s dietary inflammatory potential using scoring algorithm. It has been construct validate against various inflammatory markers, including C-reactive protein (CRP)^4,5^, tumor necrosis factor alpha^6,7^, interleukin-6^7^. Moreover, the DII has been shown to be associated with a range of other inflammatory-related conditions including diabetes^8^, cancers^9^, cardiovascular disease^10^, telomere length^11^, chronic kidney disease^12^, frailty^13^, and mortality^14^.

However, there is limited evidence regarding the potential association between DII and urinary incontinence. Therefore, we aim to investigate the cross-sectional association between DII and urinary incontinence using the National Health and Nutrition Examination Survey (NHANES).

## Methods

### Study population

Data from individuals participating in the National Health and Nutritional Examination Surveys (NHANES) (http://www.cdc.gov/nchs/nhanes.htm) were used. NHANES data are cross sectional surveys of the nationally representative, non-institutionalized US population that are sampled in two-year cycles using a complex, stratified, multi-stage, probability cluster design. Questionnaire information is obtained from an in-home interview. Participants are then invited for further examination in their mobile examination clinic (MEC) where standardized physical examination and further questioning conducted.

To increase the sample size we combined the nine survey periods from 1999 to 2016. The study population was restricted to women, age between 20 and 65 years. We excluded the individuals without complete information on UI (n = 4419), missing single 24-h dietary recall data (n = 1398) and missing data for covariate (n = 52) (see Supplementary Fig. S1 online). The National Centers for Health Statistics ethics review board approved the protocol and all participants provided written informed consent. All methods were performed in accordance with the relevant guidelines and regulations.

### Urinary incontinence

Questions on urinary incontinence were only asked of the 20 years old or older in MECs (Mobile Examination Centers). If participants answered yes to this question, “During the past 12 months, have you leaked or lost control of even a small amount of urine with activity like coughing, lifting, or exercise?” categorized as stress UI. Urgency UI was defined based on a positive answer to: “ During the past 12 months, have you leaked or lost control of even a small amount of urine with an urge or pressure to urinate and you couldn’t get to the toilet fast enough?”. Participants were considered mixed UI if they responded yes to both the stress and urgency UI questions.

### Dietary inflammation index

Dietary information were obtained from 24-h dietary recall interviews (24HRs), and then processed by using the USDA’s Food and Nutrient Database for Dietary Studies (FNDDS) to obtain micro and macronutrients. The 24HR-derived dietary data were used to calculate DII scores for all participants. The DII food parameters available in NHANES database included fat; protein; carbohydrates; vitamins A, B1, B2, B6, B12, C, D, E; niacin; grams of alcohol; omega3 and omega6 polyunsaturated fatty acids; fiber; cholesterol; saturated, monounsaturated, and polyunsaturated fatty acids; folic acid; Fe; Mg; Zn; Se; β-carotene and caffeine. Higher DII scores indicate more pro-inflammatory diets and more negative values are more anti-inflammatory. The development and validation of the DII have been discussed in detail elsewhere^15^.

### Covariates

The other possible covariates used for adjustment including age, self-reported race/ethnicity, which was then categorized as non-Hispanic white, non-Hispanic black, Hispanic (including Mexican–American) and other ethnicity. Wealth was assessed using the ratio of family income to poverty [i.e., Poverty Income Ratio (PIR)] which was calculated based on the poverty guidelines of the Department of Health and Human Services. Education was categorized as less than high school, high school/equivalent, or greater than high school. Body mass index was calculated as kg/m^2^ and as less than 25.0 (underweight/normal weight), 25.0 to 29.9 (overweight), and 30.0 or more (obese). We used composite cardiovascular disease risk score which aggregating several risk factors including, hypertension, coronary artery disease and/or history of transient ischemic attack or stroke and diabetes. The score ranges from 0 to 5. Participants were categorized as menopausal if they reported no menstrual periods because of menopause. Parity was defined as the number of total cesarean and vaginal deliveries. Other health related variables included cigarette consumption (whether smoked at least 100 cigarettes in lifetime), alcohol consumption and physical activity. All covariates were chosen based on known or suspected confounders of the relationship between DII and urinary incontinence in women.

### Statistical analysis

Data are presented as mean ± SD or proportions. Kruscal Whallis H test (continuous variables) and chi-square tests (categorical variables) were used to determine if there were any statistical differences among different DII groups (quartiles). We used multivariate linear regression model to evaluate the associations between DII and UI. According to the recommendation of STROBE statement^16^, we showed unadjusted, minimally adjusted and fully adjusted results simultaneously. Generalized additive model (GAM) was applied to adjust for age (smooth), energy (smooth) and deliveries (smooth) in all models to account for potential nonlinearity in the association between these continuous variables and UI. The subgroup analyses were performed using stratified linear regression models. The modification and interaction of subgroup were inspected by the likelihood ration test. All analyses were performed using the statistical software packages R (http://www.R-project.org, The R Foundation) and EmpowerStats (http://www.empowerstats.com, X&Y Solutions, Inc., Boston, MA). P-values were two sided with a significance level of < 0.05.

## Results

### Baseline characteristics

A total of 13,441 women age between 20 and 65 years were included in the final analysis. Of these women 3230 (24.03%) complained of UUI, 5276 (39.25%) complained of SUI and 2028 (15.09%) complained of MUI. The DII score in this study ranged from a maximally pro-inflammatory score of + 5.33 to maximally anti-inflammatory score of − 4.81. Supplementary Table S1 online displays population characteristics by DII quartiles. Those in the DII quartile 4 were more likely to be younger, non-Hispanic Black, and have lower energy intakes, lower alcohol intake per week, lower family income, lower educational attainment, higher BMI, and higher proportion of living alone, less physical activity, higher proportion of menopause, more cigarettes, more deliveries, and were more risky to CVD compared to those in DII quartile 1.

### Associations of dietary inflammation index with urinary incontinence

ORs for UI by quartile categories and one-unit increment in DII score are shown in Table 1. On multivariate analysis, when analyzing DII as a continuous variable, DII was not statistically significantly associated with UUI in crude model (OR 1.02, 95% CI 1.00–1.04, P = 0.094). However, after adjusting for age, race, energy, a one-unit increase in DII (more pro-inflammatory) was associated with significantly greater odds of UUI (OR 1.08, 95% CI 1.06–1.11, P < 0.001). After full adjustment for all covariates, the OR was slightly attenuated (OR 1.04, 95% CI 1.01–1.06, P = 0.017). Similar results were observed in SUI (OR 1.03, 95% CI 1.01–1.06, P = 0.011) and MUI (OR 1.04, 95% CI 1.00–1.07, P = 0.034). Analysis with DII categorized as quartiles revealed significantly increase odds of UUI in quartile 4 (most pro-inflammatory) compared to quartile 1(most anti-inflammatory) (OR 1.24, 95% CI 1.07–1.44, P = 0.004 for trend) in full adjustment model. In SUI and MUI, significantly associations with DII were observed when comparing participants in the highest versus the lowest DII group quartile. (OR 1.14, 95% CI 1.00–1.30, P = 0.021 for trend; OR 1.20, 95% CI 1.02, − 1.43, P = 0.022 for trend, respectively).

**Table 1:** Relationship between DII and urinary incontinence in the unadjusted, minimally adjusted and fully adjusted logistic regression models.

	Quartile of DII				P value for trend	DII (continuous)
	1	2	3	4		
	− 4.81 to 0.61	0.61–2.11	2.11–3.27	3.27–5.33		
**UUI (OR, 95%CI, P)**						
Model1	Ref	1.06 (0.95, 1.18) 0.328	1.08 (0.96, 1.21) 0.187	1.11 (1.00, 1.25) 0.06	0.055	1.02 (1.00, 1.04) 0.094
Model2	Ref	1.17 (1.04, 1.32) 0.010	1.31 (1.15, 1.48) < 0.001	1.53 (1.33, 1.76) < 0.001	< 0.001	1.08 (1.06, 1.11) < 0.001
Model3	Ref	1.07 (0.94, 1.20) 0.308	1.13 (0.99, 1.28) 0.066	1.24 (1.07, 1.44) 0.003	0.004	1.04 (1.01, 1.06) 0.017
**SUI (OR, 95%CI, P)**						
Model1	Ref	0.98 (0.89, 1.08) 0.62	0.94 (0.85, 1.04) 0.232	0.82 (0.74, 0.91) < 0.001	0.002	0.97 (0.95, 0.99) 0.001
Model2	Ref	1.15 (1.04, 1.28) 0.009	1.26 (1.13, 1.41) < 0.001	1.30 (1.14, 1.47) < 0.001	< 0.001	1.07 (1.04, 1.09) < 0.001
Model3	Ref	1.08 (0.97, 1.21) 0.138	1.15 (1.02, 1.28) 0.019	1.14 (1.00, 1.30) 0.05	0.021	1.03 (1.01, 1.06) 0.011
**MUI (OR, 95%CI, P)**						
Model1	Ref	1.02 (0.90, 1.17) 0.732	1.05 (0.92, 1.20) 0.474	1.03 (0.90, 1.18) 0.660	0.565	1.01 (0.98, 1.03) 0.540
Model2	Ref	1.20 (1.05, 1.39) 0.01	1.39 (1.20, 1.61) < 0.001	1.57 (1.34, 1.85) < 0.001	< 0.001	1.10 (1.07, 1.14) < 0.001
Model3	Ref	1.08 (0.93, 1.24) 0.318	1.16 (1.00, 1.35) 0.056	1.20 (1.02, 1.43) 0.033	0.022	1.04 (1.00, 1.07) 0.034

Model1: adjust for: None.

Model2: adjust for: age (Smooth); Race; Energy (Smooth).

Model3: adjust for: age (Smooth); Race; Energy (Smooth); BMI; menopause; alcohol intake per week; CAD score; physical activity; least 100 cigarettes; Ratio of family income to poverty; education; Marital; deliveries (Smooth).

DII, dietary inflammatory index; UUI, Urge Urinary Incontinence; SUI, stress urinary incontinence; MUI, mixed urinary incontinence; CI, confidence interval; Ref, Reference; OR, odds ratio.

### Subgroup analysis

In stratified analyses for UUI with a one-unit increment in DII score, test for interactions were significant for alcohol consumption. (P for interaction = 0.012), while the test for interactions were not statistically significant for age, race, energy, BMI, menopause, CAD score, physical activity, least 100 cigarettes, PIR, education, marital status, deliveries (see Supplementary Table S2 online). In stratified analyses for SUI, stronger positive associations were found in those who have vigorous physical activity, at least 100 cigarettes consumption and in postmenopausal women (P for interaction = 0.046, 0.003 and 0.027 respectively) (see Supplementary Table S3 online). No significant interactions were seen for other covariates on the associations between SUI and DII. Alcohol and cigarettes consumption, education attainment seems to be effect modification factors on the association between MUI and DII (P for interaction = 0.022, 0.011 and 0.035 respectively) (see Supplementary Table S4 online).

## Discussion

In this cross-sectional study using a nationally representative sample of women in the United States the women age between 20 and 65 years with more pro-inflammatory diets, as indicated by higher DII scores, are associated with an increased likelihood of UUI, SUI and MUI after adjusting for demographic and health-related covariates.

Several researches have focused on the associations between inflammatory and overactive bladder (OAB). UUI is a common symptom of overactive bladder (OAB) syndrome. Previous studies indicated a prevalence of 82.9% of UUI in OAB patients^17^. One study examined a large population among men and women aged 30–79 years from the city of Boston. Results showed a consistent association of increasing CRP levels and OAB among both men and women^18^. Results from the Boston Area Community Health study also showed a significant association of CRP levels with LUTS (including UUI and OAB) in women^19^. Several small clinical studies have also revealed a potential role of inflammation in OAB in women^20,21^. Unfortunately, very limited studies have explored the association between inflammatory potential and SUI or MUI in women directly. One study compared the serum proteomic profile in patients with SUI (n = 19) with healthy controls (n = 19), results showed that 33 proteins were induced in SUI sample which involved in inflammatory response, response to coagulation, cellular stress and cytoskeleton stability/motility^22^.

There are many possible explanations for the increased odds of UI among participants with higher DII scores. As we mentioned before, high DII score (representing pro-inflammatory diets) has been positively associated with increased levels of inflammatory markers including CRP, TNF-α, IL-6. Studies indicated that inflammatory cytokines play a key role in the modulation of connexins expression and the pathogenesis of urinary bladder dysfunction^23,24^ and inflammatory cytokines were involved in an interaction of overactive parasympathetic and peptidergic/sensory innervations of the bladder with local immune cells^21^. Furthermore, several molecular pathways indicated that inflammation accompanies the changes of SUI in rat model system. SMAD2 is a known downstream mediator of TGF-β, which plays an important role in tissue inflammation and other disorders, SMAD2 was found upregulated in SUI compared with healthy controls in previous study^25^.

To our knowledge, our study is first to use a food-based DII score which is considered associated with inflammatory potential to associate with likelihood of UI based on a nationally representative sample. We found that more pro-inflammatory diets are associated with a greater likelihood of any type of UI while controlling for key confounders. As lifestyle and behavioral treatment is considered first-line treatment for the most common types of UI^26^, our findings could be a potential therapeutic implications regarding to lifestyle such as diet. Our study has several limitations. Notably, causality could not be determined as our study is cross-sectional. Secondly, several variables of our study were self-reported and collected in questionnaire format, raising concerns for recall bias and misclassification bias, since there is possibility for respondents cannot accurately discriminate between UUI and SUI. Thirdly, although a previous study reported accurate responses to incontinence questions nearly identical to those in the NHANES questionnaire; the questionnaires using in the NHANES to identify different UI types have not validated yet; hence we need future studies to validate these questionnaires and confirm our results. Additionally, whether severity of any UI types is associated with DII scores is needed to be identified in the future studies.

## Conclusions

Our study shows that more pro-inflammatory diets, as presented by higher DII scores is associated with an increased likelihood of UI in American women younger than 65 years, suggesting inflammation as a potential mechanism linking dietary patterns and UI development. Future studies, especially those with prospective designs, are needed to strengthen our findings, explore the possible pathophysiological mechanism which could provide us more comprehensive understanding of onset and progression of UI and evaluate the potential therapeutic implications.

## Supplementary Information

Supplementary Information.

## Data Availability

Data described in the manuscript, code book, and analytic code will be made publicly and freely available without restriction at http://www.cdc.gov/nchs/nhanes.htm.
