# Supplementary material for: Associations between the dietary inflammatory index and urinary incontinence among women younger than 65 years
Source: Sci Rep. 2021 Apr 29;11:9340. doi: 10.1038/s41598-021-88833-0 (PMC8084978; doi:10.1038/s41598-021-88833-0)
Supplement: Supplementary file 1 — Supplementary Information. [file 41598_2021_88833_MOESM1_ESM.pdf]

Associations between the Dietary Inflammatory Index and Urinary Incontinence among Women younger than 65 years

Shiyu Zhang[1*], Haiyang Bian[2*], Shi Qiu[1,3*], Boyu Cai[1], Kun Jin[1], Xiaonan Zheng[1], Jiakun Li[1], Xiang Tu[1], Jianzhong Ai[1], Lu Yang[1#], Qiang Wei[1#]

*These authors have contributed equally to this work

1.Department of Urology, Institute of Urology and National Clinical Research Center for Geriatrics, West China Hospital of Sichuan University, Chengdu, Sichuan Province, China

2.Peking University School of Public Health, Institute of Reproductive and Child Health and Department of Epidemiology and Biostatistics, Beijing, China.

3.Center of Biomedical big data, West China Hospital, Sichuan University, Chengdu, Sichuan Province, China

#Correspondence to:

Prof. Qiang Wei, Department of Urology, Institute of Urology, West China Hospital of Sichuan University, No. 37, Guoxue Alley, Chengdu, Sichuan, P.R. China; Post Code: 610041; weiqiang163163@163.com, telephone: +86 18980601425

Prof. Lu Yang, Department of Urology, Institute of Urology, West China Hospital of Sichuan University, No. 37, Guoxue Alley, Chengdu, Sichuan, P.R. China; Post Code: 610041; wycleflue@163.com, telephone: +86 13541235213

Supplementary Figure S1. NHANES 1999-2016 analytic sample flow chart.

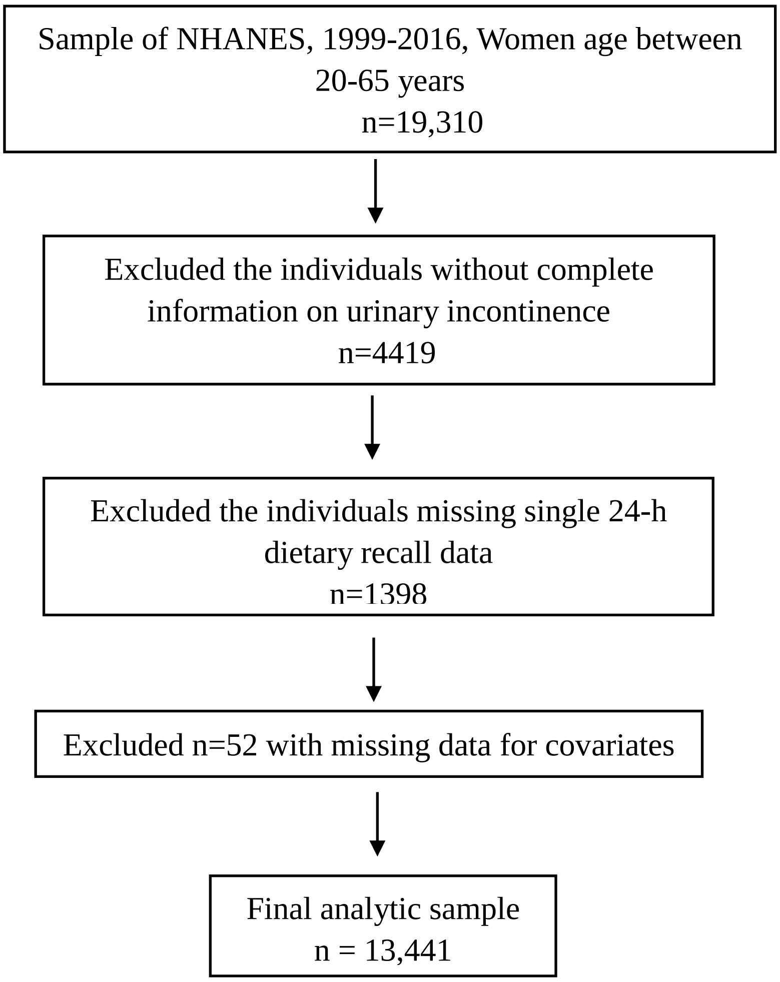

Supplementary Table S1. Baseline population characteristics according to dietary inflammatory index quartiles, National Health and Nutrition Examination Survey (NHANES), 1999–2016 (n=13,441)

characteristics	Frequency (%) or Mean ± SD				P-value
	Quartile1(n=3360) -4.81-0.61	Quartile2(n=3360) 0.61-2.11	Quartile3(n=3360) 2.11-3.27	Quartile4(n=3361) 3.27-5.33	
Age(years)	43.26± 12.67	42.67 ±12.95	42.08± 13.01	41.89 ±13.29	<0.001
Energy (kcal)	2405.95± 873.16	1996.88 ±650.71	1712.70 ±555.62	1300.37 ±547.48	<0.001
Race					<0.001
Mexican American	663 (19.73%)	661 (19.67%)	569 (16.93%)	523 (15.56%)	
Other Hispanic	305 (9.08%)	325 (9.67%)	315 (9.38%)	302 (8.99%)	
Non-Hispanic White	1437 (42.77%)	1359 (40.45%)	1427 (42.47%)	1421 (42.28%)	
Non-Hispanic Black	571 (16.99%)	715 (21.28%)	818 (24.35%)	913 (27.16%)	
Other Race	384 (11.43%)	300 (8.93%)	231 (6.88%)	202 (6.01%)	
PIR					<0.001
<1.3	831 (24.73%)	908 (27.02%)	1027 (30.57%)	1253 (37.28%)	
1.3-3.5	1232 (36.67%)	1386 (41.25%)	1372 (40.83%)	1357 (40.37%)	
>3.5	1297 (38.60%)	1066 (31.73%)	961 (28.60%)	751 (22.34%)	
Education level					<0.001
Less than high school	590 (17.56%)	740 (22.02%)	768 (22.86%)	905 (26.93%)	
High school or GED	572 (17.02%)	698 (20.77%)	808 (24.05%)	880 (26.18%)	
Above high school	2198 (65.42%)	1922 (57.20%)	1784 (53.10%)	1576 (46.89%)	
Marital status					<0.001
Married or living with partner	2225 (66.22%)	2158 (64.23%)	2086 (62.08%)	1933 (57.51%)	
Living alone	1135 (33.78%)	1202 (35.77%)	1274 (37.92%)	1428 (42.49%)	
BMI (kg/m^2)					<0.001
<25	1221 (36.34%)	1049 (31.22%)	983 (29.26%)	948 (28.21%)	
>=25, <30	899 (26.76%)	951 (28.30%)	955 (28.42%)	923 (27.46%)	
>=30	1240 (36.90%)	1360 (40.48%)	1422 (42.32%)	1490 (44.33%)	
CVD risk score					<0.001
0	2360 (70.24%)	2216 (65.95%)	2214 (65.89%)	2175 (64.71%)	

1	670 (19.94%)	753 (22.41%)	726 (21.61%)	742 (22.08%)	
2-5	330 (9.82%)	391 (11.64%)	420 (12.50%)	444 (13.21%)	
Alcohol intake per week					<0.001
never	446 (13.27%)	471 (14.02%)	551 (16.40%)	620 (18.45%)	
Up to once	2292 (68.21%)	2395 (71.28%)	2340 (69.64%)	2445 (72.75%)	
2-3 times	370 (11.01%)	303 (9.02%)	330 (9.82%)	197 (5.86%)	
4-6 times	168 (5.00%)	126 (3.75%)	87 (2.59%)	57 (1.70%)	
Daily or more	84 (2.50%)	65 (1.93%)	52 (1.55%)	42 (1.25%)	
Physical activity					<0.001
Less than moderate	1321 (39.32%)	1561 (46.46%)	1637 (48.72%)	1826 (54.33%)	
Moderate	506 (15.06%)	602 (17.92%)	567 (16.88%)	565 (16.81%)	
Vigorous	1533 (45.62%)	1197 (35.62%)	1156 (34.40%)	970 (28.86%)	
Menopause					0.008
no	2514 (74.82%)	2474 (73.63%)	2487 (74.02%)	2396 (71.29%)	
yes	846 (25.18%)	886 (26.37%)	873 (25.98%)	965 (28.71%)	
Smoked at least 100 cigarettes lifetime					<0.001
no	2262 (67.32%)	2194 (65.30%)	2027 (60.33%)	1870 (55.64%)	
Yes	1098 (32.68%)	1166 (34.70%)	1333 (39.67%)	1491 (44.36%)	
Deliveries	1.91 ± 1.54	2.01 ± 1.60	2.05 ± 1.62	2.15 ± 1.66	<0.001
UUI					0.293
no	2589 (77.05%)	2555 (76.04%)	2543 (75.68%)	2524 (75.10%)	
yes	771 (22.95%)	805 (23.96%)	817 (24.32%)	837 (24.90%)	
SUI					<0.001
no	1985 (59.08%)	2005 (59.67%)	2033 (60.51%)	2142 (63.73%)	
yes	1375 (40.92%)	1355 (40.33%)	1327 (39.49%)	1219 (36.27%)	
MUI					0.914
no	2864 (85.24%)	2854 (84.94%)	2843 (84.61%)	2852 (84.86%)	
yes	496 (14.76%)	506 (15.06%)	517 (15.39%)	509 (15.14%)	

Abbreviations: BMI, Body Mass Index; UUI, Urge Urinary Incontinence; SUI, stress urinary incontinence; MUI, mixed urinary incontinence; GED, General educational development; PIR, Poverty Income Ratio; CVD, cardiovascular disease.

Supplemental table 1. Effect size of DII on UUI in prespecified and exploratory subgroups

characteristics	NO. of participants	OR (95%CI)	P for interaction

Age (year)			0.357
<40	5628	1.00 (0.96, 1.04)	
40-65	7813	1.02 (0.99, 1.05)	
Race			0.524
Mexican American	2416	1.02 (0.96, 1.08)	
Other Hispanic	1247	1.07 (0.98, 1.15)	
Non-Hispanic White	5644	1.05 (1.01, 1.09)	
Non-Hispanic Black	3017	1.01 (0.97, 1.06)	
Other Race	1117	0.99 (0.91, 1.07)	
Energy(Kcal)			0.66
low	6718	1.01 (0.97, 1.05)	
High	6723	1.02 (0.99, 1.05)	
BMI			0.128
<25	4201	1.06 (1.01, 1.12)	
25-30	3728	1.05 (1.00, 1.10)	
⩾30	5512	1.01 (0.97, 1.05)	
Menopause			0.085
no	9871	1.02 (0.99, 1.05)	
yes	3570	1.06 (1.02, 1.11)	
Alcohol intake per week			0.012
never	2088	0.98 (0.92, 1.03)	
Up to once	9472	1.03 (1.00, 1.06)	
2-3 times	1200	1.13 (1.04, 1.23)	
4-6 times	438	1.14 (1.01, 1.28)	
daily or more	243	1.14 (0.97, 1.33)	
CVD risk score			0.335
0	8965	1.04 (1.01, 1.08)	
1	2891	1.04 (0.99, 1.10)	
2-5	1585	0.99 (0.93, 1.05)	
Physical activity			0.601
Less than moderate	6345	1.04 (1.01, 1.08)	
Moderate	2240	1.01 (0.95, 1.07)	
Vigorous	4856	1.04 (0.99, 1.08)	
Smoked at least 100 cigarettes lifetime			0.173
no	8353	1.02 (0.99, 1.06)	
yes	5088	1.06 (1.01, 1.10)	
PIR			0.992
<1.3	4019	1.04 (0.99, 1.08)	
1.3-3.5	5347	1.03 (0.99, 1.08)	
⩾3.5	4075	1.04 (0.99, 1.08)	
Education			0.320
Less than high school	3003	1.07 (1.01, 1.12)	
High school or GED	2958	1.04 (0.99, 1.10)	

Above high school	7480	1.02 (0.99, 1.06)	
Marital status			0.491
Married or living with partner	8402	1.04 (1.01, 1.08)	
Living alone	5039	1.03 (0.99, 1.07)	
Deliveries			0.738
<2	4912	1.03 (0.99, 1.08)	
≧2	8529	1.04 (1.00, 1.07)	

Above model adjusted for age (Smooth); Race; Energy (Smooth); BMI; menopause; alcohol intake per week; CVD risk score; physical activity; least 100 cigarettes; PIR ; education; Marital; deliveries (Smooth). In each case, the model is not adjusted for the stratification variable.

Supplementary table S2. Effect size of DII on UUI in prespecified and exploratory subgroups

characteristics	NO. of participants	OR (95%CI)	P for interaction
Age (year)			0.357
<40	5628	1.00 (0.96, 1.04)	
40-65	7813	1.02 (0.99, 1.05)	
Race			0.524
Mexican American	2416	1.02 (0.96, 1.08)	
Other Hispanic	1247	1.07 (0.98, 1.15)	
Non-Hispanic White	5644	1.05 (1.01, 1.09)	
Non-Hispanic Black	3017	1.01 (0.97, 1.06)	
Other Race	1117	0.99 (0.91, 1.07)	
Energy(Kcal)			0.66
low	6718	1.01 (0.97, 1.05)	
High	6723	1.02 (0.99, 1.05)	
BMI			0.128
<25	4201	1.06 (1.01, 1.12)	
25-30	3728	1.05 (1.00, 1.10)	
⩾30	5512	1.01 (0.97, 1.05)	
Menopause			0.085
no	9871	1.02 (0.99, 1.05)	
yes	3570	1.06 (1.02, 1.11)	
Alcohol intake per week			0.012
never	2088	0.98 (0.92, 1.03)	
Up to once	9472	1.03 (1.00, 1.06)	
2-3 times	1200	1.13 (1.04, 1.23)	
4-6 times	438	1.14 (1.01, 1.28)	
daily or more	243	1.14 (0.97, 1.33)	
CVD risk score			0.335
0	8965	1.04 (1.01, 1.08)	
1	2891	1.04 (0.99, 1.10)	
2-5	1585	0.99 (0.93, 1.05)	
Physical activity			0.601
Less than moderate	6345	1.04 (1.01, 1.08)	
Moderate	2240	1.01 (0.95, 1.07)	
Vigorous	4856	1.04 (0.99, 1.08)	
Smoked at least 100 cigarettes lifetime			0.173
no	8353	1.02 (0.99, 1.06)	
yes	5088	1.06 (1.01, 1.10)	
PIR			0.992
<1.3	4019	1.04 (0.99, 1.08)	
1.3-3.5	5347	1.03 (0.99, 1.08)	
⩾3.5	4075	1.04 (0.99, 1.08)	
Education			0.320

Less than high school	3003	1.07 (1.01, 1.12)	
High school or GED	2958	1.04 (0.99, 1.10)	
Above high school	7480	1.02 (0.99, 1.06)	
Marital status			0.491
Married or living with partner	8402	1.04 (1.01, 1.08)	
Living alone	5039	1.03 (0.99, 1.07)	
Deliveries			0.738
<2	4912	1.03 (0.99, 1.08)	
≧2	8529	1.04 (1.00, 1.07)	

Above model adjusted for age (Smooth); Race; Energy (Smooth); BMI; menopause; alcohol intake per week; CVD risk score; physical activity; least 100 cigarettes; PIR ; education; Marital; deliveries (Smooth). In each case, the model is not adjusted for the stratification variable.

Supplementary table S3. Effect size of DII on SUI in prespecified and exploratory subgroups

characteristic	NO. of participants	OR (95%CI)	P for interaction
Age (year)			0.696
<40	5628	1.02 (0.98, 1.06)	
40-65	7813	1.01 (0.98, 1.04)	
Race			0.291
Mexican American	2416	1.00 (0.95, 1.05)	
Other Hispanic	1247	0.97 (0.91, 1.04)	
Non-Hispanic White	5644	1.05 (1.01, 1.08)	
Non-Hispanic Black	3017	1.04 (0.99, 1.09)	
Other Race	1117	1.03 (0.96, 1.10)	
Energy(Kcal)			0.553
low	6718	0.99 (0.96, 1.03)	
High	6723	1.01 (0.98, 1.04)	
BMI			0.335
<25	4201	1.03 (0.99, 1.07)	
25-30	3728	1.06 (1.02, 1.10)	
⩾30	5512	1.02 (0.99, 1.06)	
Menopause			0.027
no	9871	1.02 (0.99, 1.05)	
yes	3570	1.07 (1.03, 1.11)	
Alcohol intake per week			0.33
never	2088	1.02 (0.97, 1.07)	
Up to once	9472	1.03 (1.00, 1.06)	
2-3 times	1200	1.10 (1.03, 1.17)	
4-6 times	438	1.07 (0.96, 1.18)	
daily or more	243	1.08 (0.94, 1.25)	
CVD risk score			0.172
0	8965	1.03 (1.00, 1.06)	
1	2891	1.07 (1.02, 1.12)	
2-5	1585	1.01 (0.95, 1.07)	
Physical activity			0.046
Less than moderate	6345	1.01 (0.98, 1.05)	
Moderate	2240	1.02 (0.97, 1.08)	
Vigorous	4856	1.07 (1.03, 1.10)	
Smoked at least 100 cigarettes lifetime			0.003
no	8353	1.01 (0.98, 1.04)	
yes	5088	1.08 (1.04, 1.11)	
PIR			0.652
<1.3	4019	1.05 (1.01, 1.09)	
1.3-3.5	5347	1.03 (0.99, 1.07)	
⩾3.5	4075	1.03 (0.99, 1.07)	
Education			0.915

Less than high school	3003	1.04 (0.99, 1.09)	
High school or GED	2958	1.04 (0.99, 1.09)	
Above high school	7480	1.03 (1.00, 1.06)	
Marital status			0.835
Married or living with partner	8402	1.03 (1.00, 1.06)	
Living alone	5039	1.04 (1.00, 1.08)	
Deliveries			0.413
<2	4912	1.05 (1.01, 1.09)	
≧2	8529	1.03 (1.00, 1.06)	

Above model adjusted for age (Smooth); Race; Energy (Smooth); BMI; menopause; alcohol intake per week; CVD risk score; physical activity; least 100 cigarettes; PIR ; education; Marital; deliveries (Smooth). In each case, the model is not adjusted for the stratification variable.

Supplementary table S4. Effect size of DII on MUI in prespecified and exploratory subgroups

characteristic	NO. of participants	OR (95%CI)	P for interaction
Age (year)			0.413
<40	5628	1.00 (0.95, 1.06)	
40-65	7813	1.03 (0.99, 1.07)	
Race			0.47
Mexican American	2416	1.02 (0.96, 1.09)	
Other Hispanic	1247	1.05 (0.96, 1.16)	
Non-Hispanic White	5644	1.06 (1.01, 1.11)	
Non-Hispanic Black	3017	1.01 (0.95, 1.07)	
Other Race	1117	0.98 (0.89, 1.08)	
Energy(Kcal)			0.88
low	6718	1.01 (0.96, 1.06)	
High	6723	1.00 (0.97, 1.04)	
BMI			0.656
<25	4201	1.05 (0.99, 1.12)	
25-30	3728	1.05 (0.99, 1.11)	
⩾30	5512	1.02 (0.98, 1.07)	
Menopause			0.146
no	9871	1.02 (0.98, 1.06)	
yes	3570	1.07 (1.02, 1.12)	
Alcohol intake per week			0.022
never	2088	0.99 (0.92, 1.05)	
Up to once	9472	1.03 (0.99, 1.07)	
2-3 times	1200	1.16 (1.05, 1.28)	
4-6 times	438	1.18 (1.02, 1.37)	
daily or more	243	1.09 (0.90, 1.31)	
CVD risk score			0.359
0	8965	1.04 (0.99, 1.08)	
1	2891	1.07 (1.01, 1.13)	
2-5	1585	1.01 (0.94, 1.08)	
Physical activity			0.555
Less than moderate	6345	1.03 (0.99, 1.08)	
Moderate	2240	1.01 (0.94, 1.09)	
Vigorous	4856	1.06 (1.01, 1.11)	
Smoked at least 100 cigarettes lifetime			0.011
no	8353	1.01 (0.97, 1.05)	
yes	5088	1.08 (1.03, 1.13)	
PIR			0.63
<1.3	4019	1.06 (1.00, 1.11)	
1.3-3.5	5347	1.03 (0.98, 1.08)	
⩾3.5	4075	1.03 (0.97, 1.08)	
Education			0.035

Less than high school	3003	1.07 (1.01, 1.14)	
High school or GED	2958	1.09 (1.02, 1.16)	
Above high school	7480	1.01 (0.97, 1.05)	
Marital status			0.149
Married or living with partner	8402	1.06 (1.01, 1.10)	
Living alone	5039	1.01 (0.97, 1.06)	
Deliveries			0.372
<2	4912	1.02 (0.96, 1.08)	
≧2	8529	1.05 (1.01, 1.09)	

Above model adjusted for age (Smooth); Race; Energy (Smooth); BMI; menopause; alcohol intake per week; CVD risk score; physical activity; least 100 cigarettes; PIR ; education; Marital; deliveries (Smooth). In each case, the model is not adjusted for the stratification variable.

STROBE Statement—Checklist of items that should be included in reports of ***cross-sectional studies***

	Item No	Recommendation	Page No
**Title and abstract**	1	(*a*) Indicate the study's design with a commonly used term in the title or the abstract	1
		(*b*) Provide in the abstract an informative and balanced summary of what was done and what was found	1
**Introduction**			
Background/rationale	2	Explain the scientific background and rationale for the investigation being reported	1-2
Objectives	3	State specific objectives, including any prespecified hypotheses	1-2
**Methods**			
Study design	4	Present key elements of study design early in the paper	2
Setting	5	Describe the setting, locations, and relevant dates, including periods of recruitment, exposure, follow-up, and data collection	2
Participants	6	(*a*) Give the eligibility criteria, and the sources and methods of selection of participants	2
Variables	7	Clearly define all outcomes, exposures, predictors, potential confounders, and effect modifiers. Give diagnostic criteria, if applicable	3
Data sources/ measurement	8*	For each variable of interest, give sources of data and details of methods of assessment (measurement). Describe comparability of assessment methods if there is more than one group	2
Bias	9	Describe any efforts to address potential sources of bias	2
Study size	10	Explain how the study size was arrived at	2
Quantitative variables	11	Explain how quantitative variables were handled in the analyses. If applicable, describe which groupings were chosen and why	2
Statistical methods	12	(*a*) Describe all statistical methods, including those used to control for confounding	3
		(*b*) Describe any methods used to examine subgroups and interactions	3
		(*c*) Explain how missing data were addressed	3
		(*d*) If applicable, describe analytical methods taking account of sampling strategy	3
		(*e*) Describe any sensitivity analyses	3
**Results**			
Participants	13*	(a) Report numbers of individuals at each stage of study—eg numbers potentially eligible, examined for eligibility, confirmed eligible, included in the study, completing follow-up, and analysed	4
		(b) Give reasons for non-participation at each stage	4
		(c) Consider use of a flow diagram	Supplemental Figure 1

Descriptive data	14*	(a) Give characteristics of study participants (eg demographic, clinical, social) and information on exposures and potential confounders	4
		(b) Indicate number of participants with missing data for each variable of interest	4
Outcome data	15*	Report numbers of outcome events or summary measures	4
Main results	16	(*a*) Give unadjusted estimates and, if applicable, confounder-adjusted estimates and their precision (eg, 95% confidence interval). Make clear which confounders were adjusted for and why they were included	4
		(*b*) Report category boundaries when continuous variables were categorized	4
		(*c*) If relevant, consider translating estimates of relative risk into absolute risk for a meaningful time period	4
Other analyses	17	Report other analyses done—eg analyses of subgroups and interactions, and sensitivity analyses	5
**Discussion**			
Key results	18	Summarise key results with reference to study objectives	5
Limitations	19	Discuss limitations of the study, taking into account sources of potential bias or imprecision. Discuss both direction and magnitude of any potential bias	6
Interpretation	20	Give a cautious overall interpretation of results considering objectives, limitations, multiplicity of analyses, results from similar studies, and other relevant evidence	5
Generalisability	21	Discuss the generalisability (external validity) of the study results	5-6
**Other information**			
Funding	22	Give the source of funding and the role of the funders for the present study and, if applicable, for the original study on which the present article is based	7
